# Superdominant right coronary artery with absent left circumflex artery

**DOI:** 10.2349/biij.7.1.e2

**Published:** 2011-01-01

**Authors:** Y Majid, M Warade, J Sinha, A Kalyanpur, T Gupta

**Affiliations:** Narayana Hrudayalaya, Bangalore, India

**Keywords:** Absent left main coronary artery, congenital defect, single coronary artery, right posterolateral ventricular branch (RPDA), atrioventricular groove, superdominant right coronary artery

## Abstract

Noninvasive imaging of coronary artery disease is rapidly replacing angiography as the first line of investigation. Multislice CT is the non-invasive modality of choice for imaging coronary artery disease and provides high speed with good spatial resolution. CT coronary angiography in addition to detecting and characterising atherosclerotic coronary artery disease is also a good imaging tool for evaluating anomalies of coronary arteries. Superdominant right coronary artery with absent left circumflex artery is one such rare coronary artery anomaly which is well evaluated with multislice CT angiography. The authors report one such case of superdominant right coronary artery with absent left circumflex artery imaged with 64-slice MDCT.

## CASE REPORT

**Figure 1:**
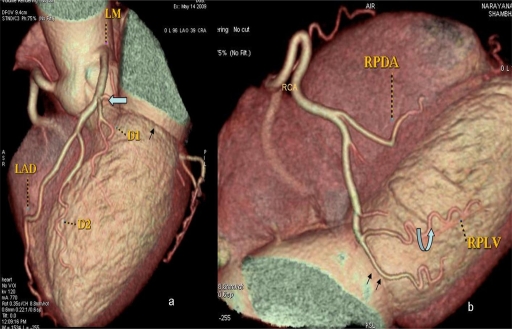
Serial axial sections of heart demonstrating absent left circumflex artery (open arrow) in the upper part of left atrioventricular groove (arrow) with left main coronary artery (LM) continuing as left anterior descending artery (LAD). Good-sized right posterolateral ventricular branch (RPLV), crossing the crux of the heart and then ascending into the inferior part of atrioventricular groove (double arrows). Several tortuous branches arising from RPLV (curved arrow) perfusing postero-lateral and lateral walls of the heart (in the usual vascular territory of LCX artery)

**Figure 2:**
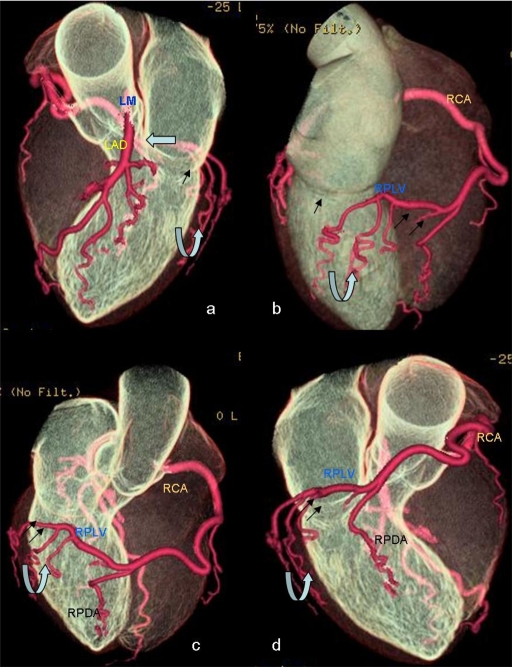
Volume rendered images of heart demonstrating absent left circumflex artery (open arrow) in the upper part of left atrioventricular groove (arrow) with left main coronary artery (LM) continuing as left anterior descending artery (LAD). Good-sized and tortuous right coronary artery (RCA). Good-sized right posterolateral ventricular branch (RPLV) arising from the right coronary artery and extended leftwards, crossing the crux of the heart and then ascending into the inferior part of atrioventricular groove (double arrows). Several tortuous branches arising from RPLV (curved arrow) perfusing postero-lateral and lateral walls of the heart (in the usual vascular territory of LCX artery).

A 55-year-old hypertensive, non-diabetic female presented to the authors’ hospital with intermittent chest pain not related to exertion. Routine blood investigations were within normal limits except for mild dyslipidemia. ECG was within normal limits; however TMT revealed mild T-wave inversion in inferior leads on mild exertion. A CT coronary angiogram was requested and performed using 64-slice GE light speed CT scanner. The CT revealed non visualised left circumflex coronary artery, likely congenitally absent (Figures 1a and 2a). The right coronary artery was good sized (Figures 1b, and 2b) with a tortuous course. The right posterolateral ventricular branch (RPLV) arising from the right coronary artery was good sized (Figure 2b) and extended leftwards, crossing the crux of the heart and then ascending into the inferior part of atrioventricular groove (Figure 1). Several tortuous branches were seen arising from it perfusing postero-lateral and lateral walls of the heart (in the usual vascular territory of LCX artery) (Figures 1 and 2).

## DISCUSSION

Conventional angiography has been the traditional gold standard for evaluating coronary artery anomalies but with the advent of multislice CT, their evaluation has become easier, non-invasive and more fascinating. Multislice CT not only allows the visualisation of these anomalies but also allows visualisation of adjacent structure thus giving a fair idea of the potential outcome. Traditionally, coronary artery anomalies have been divided into anomalies of origin, course and termination. Coronary artery anomalies have also been divided into benign and malignant types depending upon the potential clinical outcome. The anomalies of origin include multiple ostia, single coronary artery, anomalous origin of the coronary artery from the pulmonary artery and origin of the coronary artery or branch from the opposite or noncoronary sinus or from subclavian artery. The anomalies, of course, include myocardial bridging and duplication of arteries. Of greatest potential clinical concern, the arteries may have an interarterial course which may be associated with sudden cardiac death. The anomalies of termination include coronary artery fistula, coronary arcade, and extra cardiac termination.

The ideal imaging for coronary artery anomalies is angiography supported by other imaging modalities including computed tomography. Magnetic resonance angiography is very good in clearly identifying anatomy of anomalous coronary arteries because the proximal anatomy is usually unclear on coronary angiography. However, it is not good for distal course of coronary arteries. In contrast, 64-slice CT is very good in delineating these anomalies, as it has very good spatial resolution and rapid acquisition. Also, with the use of ECG gating the movement and cardiac pulsation artefacts can be minimised. The limitations relate to the administration of ionising radiation and potentially nephrotoxic or allergenic contrast agents.

Absent left circumflex coronary artery with superdominant right coronary artery is a very rare anomaly in which the left main coronary artery continues as left anterior descending artery and there is complete absence of the left circumflex artery and obtuse marginal artery. The right coronary artery is superdominant with its distal branches coursing retrogradely in the left atrioventricular groove (in the course of the normal left circumflex artery) and supplying the left ventricle. In this case, the right posterior descending artery was prominent and tortuous, and continuing retrogradely in the left atrioventricular groove.

Not many cases of this anomaly have been reported. In one case study, the patient presented with symptoms of exertional chest pain [3]. The symptoms of chest pain were thought to be due to transient ischemia of the left ventricular inferior and septal walls in conditions during which an increased oxygen demand is required. Normally, these areas are supplied by the left circumflex artery; however, in the absence of the left circumflex artery, the oxygen demand of these areas is supplied by the right coronary artery which may not be sufficient during physical exertion. So the identification of this anomaly becomes important because the symptoms may mimic atherosclerotic coronary artery disease.
